# Ectopic Nociceptor Sprouting as a Key Peripheral Driver of Pain in Rheumatoid Arthritis

**DOI:** 10.1007/s11926-025-01198-5

**Published:** 2025-07-16

**Authors:** Jayden A. O’Brien, Joseph B. Lesnak, Theodore J. Price

**Affiliations:** Department of Neuroscience and Center for Advanced Pain Studies, University of Texas at Dallas, 800 W Campbell Rd, Richardson, TX 75080 USA

**Keywords:** Inflammatory pain, Dorsal root ganglion, Nociceptor, Neuronal plasticity, Chronic pain, Transcriptomics

## Abstract

**Purpose of review:**

Pain is one of the most debilitating sequelae of rheumatoid arthritis. Established and emerging therapies offer effective disease control for many patients, though they often have underwhelming efficacy for pain relief. The uncoupling of pain intensity from disease activity and inflammation presents an ongoing challenge in both our understanding of the pathophysiology and our ability to treat joint pain. The generation of high-parameter, unbiased -omic data sets generated from patient-derived tissues is changing how we think about rheumatoid arthritis pain. In this review, we discuss the peripheral drivers of pain in rheumatoid arthritis-affected joints and their innervating primary afferents. We evaluate how human molecular immunology and neuroscience approaches are helping us unravel the heterogeneity of pain in rheumatoid arthritis and propose future directions to clarify how pain is maintained in the absence of inflammation.

**Recent findings:**

Synovial fibroblasts have emerged as key pronociceptive drivers within the rheumatic joint. Further to the classical proinflammatory mediators known to drive pain, such as cytokines and prostaglandins, bone morphogenetic proteins, ephrin signaling, and netrins appear to be upregulated in both rheumatoid arthritis-affected synovium and the innervating sensory neurons. Resulting adaptations to innervating primary afferents such as synaptogenesis and neurite outgrowth may occur in a sensory neuron subtype-specific manner causing pain that is disproportionate to inflammation.

**Summary:**

Nociceptor sprouting in the joint may explain why pain tends to persist despite adequate disease control. Future mechanistic work exploring the conditions under which these nociceptors sprout into the joint will provide new therapeutic avenues for ensuring that pain resolves alongside the inflammation associated with rheumatoid arthritis.

## Introduction

Rheumatoid arthritis (RA) is an autoimmune disease involving chronic inflammation of affected joints which progressively damages bone and cartilage. Women are 2–3 times more likely than men to have RA [1]. The precise causes of RA continue to be examined, though it is associated with autoantibodies against anti-citrullinated proteins (ACPAs) and/or rheumatoid factor (IgG) in approximately 70% of cases [2], and risk factors circulating in blood can predict onset in at-risk individuals [3]. RA involves periods of high or low disease activity: the former (active) RA is associated with macroscopic signs of inflammation in the affected joints, while in the latter these signs of inflammation are reduced or absent [4]. Disease-modifying antirheumatic drugs (DMARDs), such as methotrexate, and biologics such as tumor necrosis factor (TNF) and interleukin-6 (IL-6) inhibitors, Janus kinase (JAK) inhibitors, the CD80/CD86 inhibitor abatacept, and the anti-CD20 antibody rituximab are among current strategies to slow or halt disease progression and thereby achieve joint inflammation remission [2].

Pain is one of the classical signs of inflammation, and joint pain is the main reason why people with RA seek medical care [5]. Yet it is the aspect of disease most refractory to treatment. DMARDs, while having good success in controlling disease progression, do not consistently improve pain measures [6]. Periods of low disease activity coincide with high levels of pain in many patients [7], a phenomenon that has been referred to as residual pain [8, 9]. The presence of anti-CCP antibodies is positively associated with joint inflammation but not with levels of unacceptable pain at time of RA diagnosis [10], nor are quantitative sensory testing scores [11]. Clearly, there is a disconnect between the inflammatory drivers of RA and its associated pain.

In recognition of this disconnect, pain in the context of RA has been categorized as inflammatory or non-inflammatory [12–14]. Inflammatory pain is pain that presents in approximate proportion to the level of disease activity and signs of inflammation in the joint, and is therefore considered nociceptive; non-inflammatory pain, on the other hand, may be present when the disease is in remission, inflammation in the joint is not present, or may even initiate prior to disease onset [15]. Non-inflammatory pain in RA has been described as nociplastic [13] since it appears to arise not from the detection of noxious stimuli or damage to the nervous system, but rather due to plastic changes in the nervous system resulting from persistent stimulation of the peripheral nervous apparatus during the disease (e.g. nociceptor sensitization) [6, 16].

**Fig. 1:**
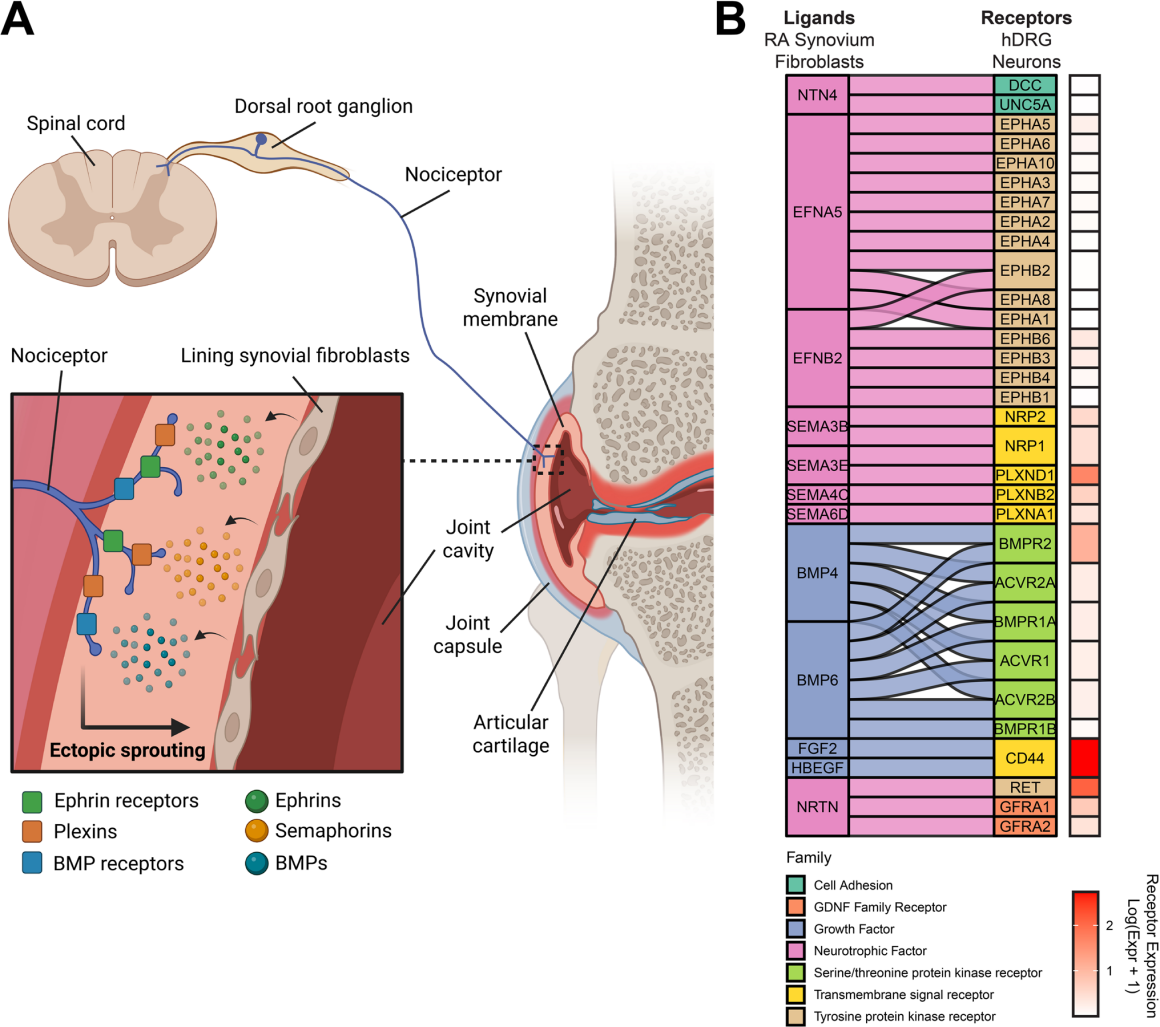
Ectopic neuronal sprouting may drive pain in rheumatoid arthritis even in states of low disease activity. (**A**) The proposed model positions lining synovial fibroblasts as the key impaired cell type in RA that drives nociceptor sprouting. These fibroblasts secrete axon guidance molecules that bind to receptors on sensory neuron nerve endings. This engages neuronal development programs and facilitates the outgrowth of these nerve endings, increasing the density of sensory innervation in the joint and extending the receptive field of these neurons into parts of the tissue that typically have little or no innervation. The identity of these sensory neurons may be putative silent nociceptors that are unsilenced by mediators in the RA joint and contribute to the sensation of pain in response to small movements. Their nociceptive signals are relayed to the spinal cord and then onward to the brain which produces the experience of pain. (**B**) Reanalysis of the ligand-receptor interactions predicted to exist between low-disease activity RA synovial fibroblasts taken from Bai et al. [28] and human dorsal root ganglion (hDRG) putative silent nociceptors. The selected ligands are upregulated in low-disease activity fibroblasts and include axon guidance, synaptogenesis, and neuronal development-related proteins. The receptors are those present in human putative silent nociceptors and their interactions with their ligands, represented by the alluvial flows, are predicted based on a previously published interactome platform for human sensory neurons [29]. The log-normalized RNA expression levels of each of these receptors in human putative silent nociceptors are represented in the heatmap. BMP: bone morphogenetic protein; GDNF: glial cell line-derived neurotrophic factor

As standardized methods for extracting cells from synovial tissue for high-dimensional analysis have emerged, and the first high-dimensional omics datasets from these tissues have been generated, our capacity to characterize the RA disease environment has expanded [17–21]. Advances in applying omics technologies to human RA tissues have updated our understanding of the mechanisms of pain in the disease. Likewise, the molecular and functional diversity of human sensory neurons has been uncovered by single-cell and spatial RNA sequencing studies [22–24]. In this review, we discuss the peripheral drivers of pain in RA, focusing on studies applying RNA sequencing to human RA tissues, including the synovium and the joint-innervating dorsal root ganglion (DRG). Based on these advances and others, we argue that in addition to peripheral and central sensitization, the sprouting of sensory neurons into the affected joint in response to the continued presence of pronociceptive and axon guidance molecules in non-inflammatory states are underappreciated drivers of RA pain (Fig. 1A). We then provide perspectives on possible avenues for future investigations and implications for therapeutic targets.

## Molecular and Cellular Pronociceptive Changes in the Synovium

It is well documented that the proinflammatory mediators present in the rheumatic joint drive pain. Proinflammatory cytokines, such as TNF, IL-1β, and IL-6, and other mediators such as prostaglandins, can directly sensitize nociceptors via receptors expressed on their nerve endings [25]. This modulates the electrophysiological properties of the sensory neuron, leading to hyperexcitability. Likewise, increased JAK/STAT signaling can drive pain through the production of proinflammatory cytokines such as type I interferons [26]. These factors have been thoroughly reviewed previously [27]. And yet, TNF and IL-6 DMARDs have limited success in bringing pain back to baseline levels [6] and pain is often present even during periods of low disease activity [7]. Since patient-reported pain symptomology does not correlate well with levels of inflammation in individuals with RA, alternate hypotheses for the processes driving pain require consideration and investigation.

To uncover synovial joint changes that could be potential mediators of patient reported symptomology, a recent article by Bai and colleagues [28] explored transcriptomic alterations in synovial tissue from individuals with either high or low synovial inflammation. In those with high synovial inflammation, there was a direct correlation between the number of cells in the synovium and pain measures; however, in those with low synovial inflammation, there was no correlation between cellular density and pain. Using bulk RNA sequencing of synovial joint tissue and a machine learning algorithm, the authors identified a group of 815 genes that were upregulated in individuals with low synovial inflammation compared to those with high synovial inflammation and were positively correlated with pain measures. Using previously reported synovial joint single cell RNA sequencing datasets, it was found that the majority of the 815 genes had the highest expression in synovial joint fibroblasts, signifying a more prominent role of fibroblasts in pain generation in those with low inflammatory rheumatoid arthritis. Interestingly, the 815 genes were enriched for pathways including nervous system development, neurogenesis and neuron differentiation, suggesting that fibroblasts could be driving changes in the peripheral nervous system to lead to pain symptomology (Table 1).

**Table 1:** Top Gene Ontology Biological Process Terms were determined with ENRICHR and ranked based on adjusted p-value for the 815 synovial tissue genes associated with pain in individuals with low inflammatory RA

GO Biological Process Term	Adjusted *P*-value
Neuron Projection Guidance (GO:0097485)	0.02
Axon Guidance (GO:0007411)	0.02
Regulation of Dendritic Spine Morphogenesis (GO:0061001)	0.02
Regulation of Cartilage Development (GO:0061035)	0.02
Negative Regulation of Membrane Protein Ectodomain Proteolysis (GO:0051045)	0.02
Regulation of Membrane Protein Ectodomain Proteolysis (GO:0051043)	0.02
Axonogenesis (GO:0007409)	0.03
Regulation of Postsynapse Organization (GO:0099175)	0.08
Cellular Response to BMP Stimulus (GO:0071773)	0.08
Regulation of Neuron Projection Development (GO:0010975)	0.08

To further explore how synovial fibroblasts could be leading to pain symptomology, we utilized a ligand-receptor interactome to look at the soluble factors that could be released from these fibroblasts and bind to receptors expressed by nociceptors [29]. Taking the 815 low synovial inflammation pain genes, we identified a total of 162 unique interactions consisting of 27 upregulated ligands and 105 receptors previously identified on human nociceptors [22, 24]. Ligand genes were enriched for biological processes of axonogenesis and axon guidance (Table 2) suggesting they could drive increased neurite growth into the joint, a common pathophysiological alteration in several musculoskeletal diseases [30–35]. Bai and colleagues focused on netrin-4, which binds to several receptors including neogenin, UNC5, and DCC which are located on nociceptors. It was shown that cultured mouse DRG calcitonin gene-related peptide (CGRP) + neurons have increased sprouting and branching when cultured with netrin-4. This demonstrates a direct potential mechanism through which synovial joint fibroblasts could be secreting netrin-4 to lead to increased neurite growth in the joints of individuals with RA to lead to pain symptomology. Given differences in netrin-4 receptors between mice and humans, we expect that this in vitro experiment may underestimate the effects of netrin-4 on human nociceptors.

**Table 2:** Top Gene Ontology Biological Process Terms were determined with ENRICHR and ranked based on adjusted p-value for the synovial tissue ligands and human DRG receptors identified in the ligand-receptor interactome generated from the 815 synovial tissue genes associated with pain in individuals with low inflammatory RA

Synovial Fibroblast Ligands		hDRG Neuron Receptors	
GO Biological Process Term	Adjusted *P*-value	GO Biological Process Term	Adjusted *P*-value
Axonogenesis (GO:0007409)	3.01E-11	Glutamate Receptor Signaling Pathway (GO:0007215)	2.67E-39
Neuron Projection Guidance (GO:0097485)	3.01E-11	Synaptic Transmission, Glutamatergic (GO:0035249)	2.89E-28
Axon Guidance (GO:0007411)	7.29E-11	Modulation of Chemical Synaptic Transmission (GO:0050804)	3.15E-24
Mesenchymal Cell Migration (GO:0090497)	4.72E-10	Cell Surface Receptor Protein Tyrosine Kinase Signaling Pathway (GO:0007169)	1.06E-23
Neural Crest Cell Migration (GO:0001755)	8.30E-10	Regulation of Synaptic Transmission, Glutamatergic (GO:0051966)	1.67E-18
Neural Crest Cell Development (GO:0014032)	2.58E-09	Chemical Synaptic Transmission (GO:0007268)	2.31E-18
+ Reg of Phosphatidylinositol 3-Kinase/Prot Kinase B Signal Transduction (GO:0051897)	3.41E-09	Ephrin Receptor Signaling Pathway (GO:0048013)	5.53E-18
Positive Regulation of Intracellular Signal Transduction (GO:1,902,533)	2.96E-08	Regulation of Trans-Synaptic Signaling (GO:0099177)	1.83E-17
Regulation of Phosphatidylinositol 3-Kinase/Protein Kinase B Signal Transduction (GO:0051896)	2.96E-08	G Protein-Coupled Glutamate Receptor Signaling Pathway (GO:0007216)	5.24E-16
Semaphorin-Plexin Signaling Pathway (GO:0071526)	1.33E-07	Neuron Projection Guidance (GO:0097485)	1.10E-15

The interactome analysis revealed several other interactions that could lead to pain symptomology in individuals with low synovial inflammation RA. This included ephrins, semaphorins, and bone morphogenetic proteins (BMPs). Ephrins and their receptors are the largest family of receptor tyrosine kinases and have gained interest as a potential therapeutic target for pain. Preclinical models demonstrate the activation of ephrins, particularly ephrin-B2, in sensory neurons mediates pain development in several pain models [36, 37]. Recently, it has been demonstrated that ephrin-B2 sensitizes both mouse and human DRGs to PGE_2_ stimulation [38]. Semaphorins are a class of proteins that signal through plexin and neuropilin receptors and are involved with guiding nerve cell growth during development [39]. Recent work suggests that semaphorins play a role in pain development via semaphorin-4C signaling through plexin B2 in sensory neurons [40]. The BMPs belong to the tumor growth factor (TGF)-β superfamily and play a role in bone formation and neurodevelopment. BMPs signal through a receptor consisting of a BMP and ACV subunit which are expressed by human nociceptors [41]. Recent findings show that the gain of function mutation in ACVR1 that causes fibrodysplasia ossificans progressive (FOP) is also directly tied to pain in the disease. The ACVR1 mutation increases sensory neuronal hyperexcitability in patient-derived induced pluripotent stem cell-derived sensory neurons, which is consistent with the idea that increased BMP to ACVR signaling could mediate development of pain symptomology in individuals with RA [42].

We have performed a reanalysis of the synovial fibroblast to DRG ligand-receptor data from this study, focusing on the receptors with the highest normalized expression level on putative silent nociceptors (Fig. 1B). We focused on this subtype of nociceptor because these neurons are known to play an important role in chronic pain in humans, and they innervate joints [43, 44]. These neurons are normally not mechanically sensitive, but can be “unsilenced” by stimulation of the neuron with certain mediators. If these neurons sprout into the synovium of the joint, they could cause this tissue to become mechanically sensitive, driving pain with small movements in the disease. In humans, they can be classified by their expression of *OSMR* and *SST* [24]. The receptors with the highest expression on silent nociceptors included *CD44*, *RET*, and *PLXND1*. The CD44 gene encodes for a cell surface receptor involved in several cellular functions such as cell migration, proliferation, and neurite growth [45]. The ligands that were predicted to be binding with CD44 were fibroblast growth factor 2 and Proheparin-binding EGF-like growth factor (HBEGF). In individuals with RA, synovial joint HBEGF + macrophages mediate the invasiveness of synovial fibroblasts and we have demonstrated that HBEGF directly activates mouse DRG neurons and causes mechanical hypersensitivity when injected into the paw [29, 46]. The gene *RET* encodes a transmembrane receptor tyrosine kinase and was predicted to be activated by the ligand neurturin (*NRTN)* but can also be bound by all GDNF family ligands including GDNF, artemin, and persephin. Activation of RET signaling requires binding of GDNF family ligands to a GDNF family receptor-α (GFRα1-4) which then forms the RET-GFRα complex to induce intracellular signaling. Activation of this pathway has been shown to directly activate rat DRG neurons, cause paw hypersensitivity, and mediate pain behaviors in a model of inflammatory-bone pain [47]. Lastly, *PLXND1* encodes for the receptor plexin-D1 and was predicted to be activated by semaphorin-3E. Again, semaphorins play an important role in mediating axon guidance and thus this interaction could also be driving alterations in axons in the joints of individuals with RA. In sum, this ligand-receptor interactome from low inflammatory synovial joints highlights numerous potential mechanisms that could be modulating pain generation in this subset of individuals. It is imperative that future work follows up on these potential mechanisms with preclinical models and functional human DRG neuronal experiments.

## Molecular and Physiological Adaptations in Sensory Neurons Innervating the Rheumatoid Arthritis Joint

Sensory neurons, which include proprioceptors, low threshold mechanoreceptors, and nociceptors, have a pseudounipolar morphology with axon terminals at the distal site (the innervating tissue) and the proximal site (the spinal cord), with the cell body residing in the dorsal root ganglion (DRG). Primary afferents are anatomically distinguished by their axon diameter and degree of myelination, and functionally distinguished by their electrophysiological and pharmacological properties [48]. The majority of primary afferents in synovial joint tissues are unmyelinated C-fibers with free nerve endings, and so nociceptors are thought to constitute most sensory neurons innervating the joint [49]. In addition to sending sensory signals toward the spinal cord, sensory neurons can also release neuropeptides from their distal terminals in the innervating tissue, causing neurogenic inflammation [50].

For example, putative silent nociceptors uniquely express the combination of *OSMR*, *CHRNA3, SST,* and *CCK* in humans and are known to innervate the joint [24, 43]. Unlike neurons of the central nervous system, peripheral neurons are capable of regeneration and can reengage neuronal development gene programs following damage. Sensory neuron ‘sprouting’, in which ectopic afferents enter tissues undergoing processes associated with damage and wound healing, can drive pain in tissues such as the bladder [51], endometrium [52], and skin [53]. It has been known for decades that the number of sensory neuron nerve endings is higher in rheumatoid arthritis-affected joints compared to both osteoarthritic and healthy joints, though the importance of these findings for our understanding of pain in RA has been long underappreciated and the molecular mechanisms poorly understood [32–34, 54].

To address this, Hall and colleagues [55] conducted bulk RNA sequencing of human lumbar DRGs from a small cohort of RA patients and healthy controls. This was conducted with the goal of characterizing alterations in the joint-innervating peripheral nervous system transcriptome that may be induced by changes in the innervated joint. They found 52 protein-coding differentially expressed genes, many of which encoded secreted proteins and those with transmembrane domains, which suggests that the strongest changes in the RA DRG involve intercellular signaling processes.

A major finding was that immunoglobulin-related genes, including the IgG genes *IGHG2* and *IGHG3*, were significantly upregulated. The same authors also observed upregulation of these genes in diabetic neuropathic pain related to type 2 diabetes, which importantly does not have a known autoimmune origin [56]. This raises the possibility that the DRG immunoglobulin signature may be associated with pain rather than merely the result of RA seropositivity. Indeed, B cells are present in the human DRG, and become more abundant in disease states associated with chronic pain [57]. IgG-FcgR binding in the DRG is associated both with autoimmune chronic pain disorders and nonautoimmune neuropathic pain [58, 59]. In fibromyalgia, autoreactive IgG binds DRG satellite glial cells in fibromyalgia [60]. Satellite glia-binding IgG could cause dysfunction in these cells and thereby dysregulate their role in modulating the firing properties of the ensheathed sensory neuron. This study was not sufficiently powered to interrogate differences between seropositive and seronegative RA patients; it will be important to specifically investigate whether seronegative individuals also have an increased peripheral nervous system B cell signature, which may suggest the generation of antibodies against alternate self- and/or neoantigens driving their pain phenotype that are distinct from the ACPAs involved in disease activity.

The authors also reported alterations in two inwardly-rectifying potassium channel-related genes. *KCNJ8* (Kir6.1, a subunit of the KATP channel) was upregulated in patients with RA, while *KCNJ1* (Kir1.1 or ROMK) was downregulated. Both of these channels are mainly expressed in human DRG non-neuronal cells, including pericytes, fibroblasts, and immune cells, but are also expressed in pruriceptors and Aβ-mechanoceptors [22]. KATP channel activity has been associated with chronic pain in other contexts, particularly migraine where activators of this channel can initiate attacks [61]. There are few reports on the role of Kir1.1 in pain, though it is similarly downregulated in rat DRG in the paclitaxel model of chemotherapy-induced neuropathic pain in which sensory neurons display spontaneous activity [62]. It will be important to resolve whether these changes in expression in RA are occurring in these sensory neurons, which would support a direct role in nociceptor spontaneous activity, or in non-neuronal cells, which may have indirect effects on sensory neuron function. Attributing these changes to specific cell types will require additional studies using single-cell techniques. There were no detected changes in voltage-gated sodium channels or acid-sensing ion channels in this small cohort, which have been observed in rodent models in RA but not yet demonstrated in humans [63, 64]. These ion channel families will be important to further investigate in order to understand the electrophysiological basis for potential spontaneous activity and peripheral sensitization in RA.

Perhaps most intriguingly, there was a clearly upregulated signature of neuronal development in this RA cohort. The changed neuronal genes were mainly surface receptors involved in axon guidance and synaptogenesis, including ephrins (*EFNB3*), growth factors (*NTRK1*), and transcription factors involved in early neuronal development and cell type specification (*PAX3, DLX5*). The upregulation of ephrin receptors, in particular, is interesting since ephrin ligands are upregulated in the low disease activity RA synovium, as previously discussed [28]. This points to a model in which ephrin upregulation in the joint drives sprouting and sensitization through ephrin B (EphB) receptors located on sensory neurons that innervate the joint, which may occur even in states of low disease activity. This ligand-receptor target should be prioritized for its potential in disease modification for pain.

What remains unclear is whether the neurons displaying a sprouting phenotype preferentially belong to a particular subclass of human sensory neurons. For example, the sprouting of silent nociceptors into the joint could provide a mechanism for persistent arthralgia in the absence of RA disease activity, but this needs to be tested experimentally using markers for this subset of nociceptor. It also remains to be seen whether these sprouting neurons indeed have alterations in their electrophysiological properties indicative of sensitization. To address these unknowns, single-cell or spatial -omics approaches applied to RA DRGs will provide crucial information on exactly which sensory neuron subtypes are dysfunctional. Then, to determine whether the sensory neurons innervating the RA joint engage in spontaneous activity, ultrasound-guided microneurography could be conducted on the joint-innervating nerves of RA patients. Finally, the sprouting factors that have been identified in the joint could be applied to nociceptors in dissociated cultures to confirm that they can induce neurite outgrowth and sensitization. It is possible that the presence of sprouting in some, but not all, individuals may underly the heterogeneity of pain phenotypes in RA. Addressing these questions may reveal therapeutic targets for persistent pain in RA that is not adequately controlled by DMARDs, and creates the possibility for a new class of DMARDs that effectively treat inflammation and pain in most RA patients.

## The Heterogeneity of Rheumatoid Arthritis Phenotypes

To determine if high disease activity levels bear a relationship to the cell types present in the human RA joint, Zhang and colleagues [65] used CITE-seq to phenotype the immune and stromal cells present in the inflamed synovium of RA patients, which builds on their previous work integrating single-cell RNA sequencing and mass cytometry data from these tissues [66]. They generated six phenotypes of RA based on the relative abundance of lymphocytes, fibroblasts, and macrophages in the synovium, termed cell type abundance phenotypes (CTAPs). Membership to these disease phenotypes predicted patient responses to biological DMARDs. For example, those with a high proportion of fibroblasts, but few lymphocytes, had poorest response to treatments in rituximab and tocilizumab trials, but those with high numbers of lymphocytes were among the best responders. The authors conclude that these represent distinct inflammatory phenotypes that are driving disease in different cohorts of patients rather than any differences in disease course or levels of activity.

Though CTAPs were not distinguishable based on disease activity scores, pain outcomes were not measured. It is therefore unclear whether some CTAPs are more likely to have persistent pain than others. One possibility that arises from recent findings is that synovial fibroblast dysfunction drives pain in patients with few infiltrating lymphocytes, while immune cells contribute to a distinct (or perhaps additive) pronociceptive phenotype that further drives pain in those individuals. In this model, biological DMARDs like TNF inhibitors would target immune cell-mediated inflammation but not underlying synovial fibroblast dysfunction that causes pain to persist even when these inflammatory states are adequately treated.

It would be informative to know whether some CTAPs are more resistant to pain treatments, and whether this relates to differences in the level of aberrant nerve sprouting in the joints between phenotypes. For example, if nociceptors are indeed sprouting in the joint in RA, does controlling the disease result in the paring back of the denser innervation? One way to measure this sprouting may be to take a synovial fluid biopsy and measure the levels of neuropeptides associated with the sensory neuron subtypes prone to sprouting; in the case of putative silent nociceptors, these would be somatostatin and cholecystokinin [24, 67]. The predicted presence of nociceptor sprouting could then be cross-referenced with the patient’s CTAP to determine if there exists a relationship. We could then ask the question, how can we prevent ectopic nociceptor innervation of joint tissues, and once established, can we remove them by attempting to modulate the cell-type phenotype?

## Impaired Inflammation Resolution as a Driver of Nociception

One idea that emerges from critically appraising the discussed evidence is that, in some RA patients, endogenous inflammation resolution mechanisms in the joint that reduce disease activity are impaired in a manner that continues to drive nociception. As a result, some individuals that experience success in disease control using DMARDs may nevertheless not see their associated pain resolve because key pronociceptive processes remain uncontrolled. Synovial lining fibroblasts are proposed as candidate cells resident in the joint that can continue to secrete pronociceptive and neurogenic mediators such as ephrins even when disease activity is low. If this is the case, the goal must be to develop strategies for switching the phenotype of these cells to one that does not express these pronociceptive and neuronal sprouting factors.

This suggested framework is based on the understanding that the resolution of inflammation is not merely the absence of proinflammatory mediators, but is rather an active and distinct process. This is clearly the case in RA given the high relapse rate following cessation of TNF inhibitors, indicating that the inhibition of pro-inflammatory signals is insufficient to completely resolve inflammation [68]. The resolution of inflammation requires the engagement of specific cellular and molecular programs [69]. These programs include pro-resolution cytokines such as IL-4, IL-9, IL-10, and IL-13; neurotrophic factors such as meteorin; neutrophil extracellular traps (NETs); regulatory T cells; and a class of lipid molecules termed specialized pro-resolving mediators (SPMs) [70–74]. The resolution of pain is a distinct process from the resolution of inflammation, though it nevertheless shares some common pathways and features [75, 76].

Many of these pro-resolution processes, particularly those associated with inflammation-induced damage, include tissue repair and wound healing programs. These programs involve the growth or repair of blood vessels (angiogenesis) and nerve endings (axonogenesis) into the regenerating tissue, processes which tend to co-occur [77–79]. Angiogenesis is a well understood contributor to the pathogenesis of RA for its ability to facilitate leukocyte infiltration into the joint. Axonogenesis requires the release of growth factors, axon guidance molecules including ephrins, and other signaling molecules required for neuronal development. When these factors continue to be released but fail to resolve to a true homeostatic state, the pro-repair processes may themselves become aberrant and promote pain rather than achieving tissue repair [80].

To test the hypothesis that impaired pro-resolution processes drive nociceptor sprouting in RA, conditioned media generated by culturing primary synovial fibroblasts from RA patients with low disease activity could be applied to dissociated human DRG neurons. Then, spontaneous activity could be measured using patch clamp electrophysiology, and the potential for ectopic sprouting tested using neurite outgrowth assays. Bai and colleagues have demonstrated that this conditioned media can induce neurite outgrowth using mouse DRG neurons [28]. These mediators are well characterized in other chronic inflammation contexts, so targeted multiplex assays could be used to identify these mediators and confirm which are most responsible for inducing sprouting. Finally, it could be investigated whether the inhibition of synovial angiogenesis also abrogates neuronal sprouting. Inhibiting vascular endothelial growth factor (VEGF) receptors and kinases, key targets of antiangiogenetic drugs in the context of cancer, have been considered for use in RA and attenuate the development pain behaviors in RA rodent models [81, 82]. These drugs could be tested in in vitro and in vivo RA models for their antinociceptive potential, which would demonstrate a role for angiogenesis in residual pain.

Another challenge for targeting impaired resolution processes therapeutically is the previously outlined heterogeneity of synovial cell type phenotypes between individuals, which may present parallel pathways by which ectopic sprouting is facilitated. A possible solution is the modulation of these other cell types that may be in turn influencing resident fibroblasts. One example is macrophages. Single-cell RNA sequencing of synovial macrophages found a role for pro-resolution CD206 + macrophages in the induction of a pro-repair phenotype in fibroblast-like synoviocytes (FLS) when they were co-cultured [83]. While pro-inflammatory macrophages induced FLSs to release proinflammatory and joint-destroying molecules such as matrix metalloproteinases and IL-6, CD206 + macrophages instead increased the expression of genes related to collagen production and TGF-β response. Repolarizing these macrophages may be one way to resolve the aberrant fibroblast phenotype. It must be said, however, that macrophages are in turn susceptible to repolarization themselves by neighboring T cells [84, 85], so lasting resolution of the phenotype may be challenging using this approach.

## Direct Therapeutic Targeting of Sensory Neurons

Given the complexities discussed above, how should we approach resolving persistent, non-inflammatory RA pain therapeutically? The most direct method would be to develop therapeutics that prevent ectopic sensory neuron sprouting by disrupting the axon guidance molecules that support their outgrowth. Targeting ephrins and their receptors is one such avenue to disrupt ectopic sprouting in the joint. Small molecule drugs and biologics against ephrin receptors are under investigation in multiple diseases [86], and tetracyclines repurposed to target the EphB1 receptor have shown promise in preclinical models of neuropathic pain [87]. Ephrin signaling may be useful for targeting established pathology, but we have also now developed methods for identifying people at-risk for developing RA. If we can prophylactically target these pronociceptive programs prior to the establishment of disease, we may have better access at achieving the partial or complete resolution of pain by minimizing the degree of ectopic sprouting in the first instance.

An alternative to targeting ephrins and their receptors themselves is inhibiting their downstream signaling mechanisms, such as mitogen-activated protein kinase interacting kinase (MNK) which is currently being assessed for neuropathic pain [88]. Other options may be drugs targeting sensory neuron ion channels, such as sodium channel blockers. The Nav1.8 inhibitor suzetrigine, for example, has been recently approved by the FDA for acute pain and is currently seeking approval for chronic pain indications.

If we wish to target the underlying pathology preventing the resolution of pain in affected individuals, targeting angiogenesis by trialing existing FDA-approved VEGFR2 inhibitors human RA patients with residual pain could be attempted, but the substantial toxicities that accompany this drug class represent a challenge. Tissue-specific drug delivery may be one way around this to restrict the drug to the affected tissue. Cell-based therapies may be worthy of investigation since these are more likely to disrupt the existing dysfunctional intercellular relationships driving pathology. Existing discussions have focused on B cell depletion using CAR T cells, but targeting other cell types, such as synovial fibroblasts and macrophages, may be considered and could be personalized to the CTAP of each individual [89].

Of the existing drugs indicated for RA, JAK inhibitors have better analgesic efficacy than other DMARDs. This may be because they have separate antinociceptive activity in addition to their disease activity-controlling mechanism. Li and colleagues observed that the sensitization of human induced pluripotent stem cell (iPSC)-derived neurons by synovial fluid taken from RA patients with high levels of disease activity was diminished by the presence of the JAK inhibitor tofacitinib [90]. The authors linked this to the presence of STAT3-related cytokines in the synovial fluid – such as leukemia inhibitory factor (LIF), IL-11, and type I interferons, but other work has also demonstrated that these drugs attenuate angiogenesis by attenuating VEGF production by synovial fibroblasts [91]. Unfortunately, the tolerability of the side effects remains an ongoing issue for these drugs.

## Conclusions

The peripheral drivers of RA pain go beyond the pronociceptive and proinflammatory mediators released by the infiltrating and resident immune and stromal cells of the affected joint. Alterations to the peripheral nociceptive apparatus, such as the sensitization of specific subsets of sensory neurons, activation of silent nociceptors, and the ectopic sprouting of sensory nerve endings into joint tissues, are additional drivers with increasing bases of evidence. However, these most recent studies into the mechanisms of human RA pain are still within the realm of discovery, and further experimental evidence is required to pinpoint exactly which of these predicted factors actually underly pain in RA and whether they offer the possibility of therapeutic development.

Disease activity continues to form the primary endpoint of investigations into RA pathophysiology and therapeutics, but a greater focus on pain outcomes will accelerate our understanding of what remains the most difficult aspect of RA to treat: pain. We propose that recent advances in this area offer a new paradigm for understanding persistent pain in RA explained by ectopic sprouting of nociceptors in the RA joint. Targets causing this pathology have been pinpointed and new therapeutics can be developed to test the hypothesis, through clinical trials, that non-resolving pain in RA is driven by ligand-receptor interactions causing neo-innervation of synovial tissues and nociceptor sensitization.

## Key References and Annotations

- Bai et al. 2024 [28]: This is the first study to demonstrate that lining fibroblasts from RA patients with low inflammation upregulate secreted factors that promote neurite outgrowth in nociceptors. It provides molecular and cellular evidence in humans for mechanisms by which pain and synovial inflammation do not correlate.
- Hall et al. 2024 [55]: The authors provide the first transcriptomic analysis of alterations in the dorsal root ganglion in rheumatoid arthritis compared to non-arthritic controls. The upregulation of genes involved in neuronal development and B cell-mediated autoimmunity provide novel potential mechanisms by which the peripheral sensory nervous system is dysregulated in the disease and contribute to pain.
- Zhang et al. 2023 [65]: This study uncovered the heterogeneous cellular phenotypes driving disease activity between individuals with RA. Fibroblasts, macrophages, and lymphocytes may each have very different levels of importance in their contribution to disease activity; this provides a useful model for thinking about how heterogeneous mechanisms may also drive pain in the absence of a pro-inflammatory state.

## Data Availability

No datasets were generated or analysed during the current study.
